# The Effect of Thermocycling on Surface Layer Properties of Light Cured Polymer Matrix Ceramic Composites (PMCCs) Used in Sliding Friction Pair

**DOI:** 10.3390/ma12172776

**Published:** 2019-08-29

**Authors:** Daniel Pieniak, Agata Walczak, Agata M. Niewczas, Krzysztof Przystupa

**Affiliations:** 1 Department of Mechanics and Machine Building, University of Economics and Innovations in Lublin, Projektowa 4, 20-209 Lublin, Poland; 2 The Main School of Fire Service, Faculty of Fire Safety Engineering, Slowackiego 52/54, 01-629 Warsaw, Poland; 3 Department of Conservative Dentistry with Endodontics, Medical University of Lublin, Karmelicka 7, 20-080 Lublin, Poland; 4 Department of Automation, Lublin University of Technology, Nadbystrzycka 36, 20-618 Lublin, Poland

**Keywords:** wear, hardness, surface layer, thermocycling, dental materials

## Abstract

This paper discusses the problem of thermocycling effect of light-curing polymer–ceramic composites. Cyclic thermal shocks were simulated in laboratory conditions. As a rule, these loads were supposed to reproduce the actual conditions of biomaterials exploitation. Periodically variable stresses occurring in dental restorations are associated with the wear of cold and hot foods and beverages. They lead to changes in the properties of composites, including the properties of the surface layer. The aim of the work was to assess the impact of cyclic hydrothermal interactions on the properties of the surface layer of composites relevant to the operational quality. Two commercial materials manufactured by the world’s leading producer (3M ESPE)—Filtek Z550, Filtek Flow and two experimental, micro-hybrid and flow type composites marked Ex-mhyb(P) and Ex-flow(P), respectively. All tests were carried out before and after hydro-thermal cycles (flowing water thermocycling). Micro-hardness test using the Vickers method, indentation hardness, and resistance to tribological wear in a ball–disc system in sliding friction conditions were performed. In addition, observations of the surface layer of composites on the SEM (scanning electron microscope) were carried out. It was noticed that semi-liquid composites, containing a smaller amount of filler, retain higher stability of mechanical and tribological properties of the surface layer under cyclic hydro-thermal loads. Coefficient of friction of samples after hydro-thermal cycles increased for micro-hybrid materials and Filtek Flow (FFlow) composite. In the case of Ex-flow(P) material, the coefficient of friction decreased. The microhardness of composites also changed, the variability of this size depended on the type of material. Composites with a higher content of filler particles were characterized by greater variability of microhardness under the influence of thermocycles. The resistance to tribological wear also changed in a similar way. Composites containing higher volume fraction of inorganic filler showed higher tribological wear after thermocycling. The wear resistance of flow composites changed to a lesser extent, after thermocycling increased. The paper also showed that, in real kinematic nodes, the surface layer of light-curing ceramic–polymer composites is exposed to significant non-tribological (erosive, thermal, and chemical) defects that synergize with tribological ones. In slip pairs loaded dynamically, under mixed friction conditions, tribological wear of PMCCs (polymer matrix ceramic composites) is manifested by spalling (spalling of the material flakes, in particular the polymer phase) and pitting (crushing wear caused by wear products, in particular large filler particles or clusters, previously adhesively extracted).

## 1. Introduction

The problem of operational (usable) durability is well established in the issues of machine operation. It is also undertaken in reference to materials and medical devices subjected to biomechanical workload [1,2,3,4,5,6,7,8,9,10]. However, understanding the importance of durability and reliability in the operation of medical facilities is not common, and durability studies are utilitarian. It is possible that the problem is undoubtedly the nuisance and significant labour-intensity of durability tests and the necessity of owning and manufacturing specialized equipment. Therefore, research in this area is often carried out by interdisciplinary teams, including researchers with experience in mechanical engineering, biomedical engineering, and dentistry [11].

The damage to the surface is of key importance for durability and reliability of medical devices. This problem lies in the interest of the scientific discipline of tribology. Currently, technical tribology covers issues of durability and reliability of friction nodes. That is why in technical tribology there are two categories-wear and lubrication [12]. Wear as a utilitarian category also applies to biotribological nodes. This is, among others, an empirical description of the wear process in a biotribological friction node using technical tribology tools. Dental biotribology deals with a number of research problems, including: roughness and enamel wear [13], tooth brushing wear [14], wear of dental composites in oral environment [15,16], and mechanical contact of dental materials against natural tooth enamel [17,18,19]. Resistance to wear determines the durability of dental restorative composites [20].

Dental materials include curing light polymer–ceramic composites (PMCCs) classified according to manufacturing technology, applications and performance [21]. These are the materials used in the reconstruction of human teeth. Reconstructions are exposed to oral environment factors. They are subjected to thermocycles [22], among others, which are the result of eating foods, including hot soups, cold and warm drinks, and ice cream. A typical temperature range on the tooth surface is in the range of 1–50 °C [23,24,25]. However, there are often higher and lower temperatures [26,27].

As emphasized in a number of works [28], the effect of thermal cycling is noticeable and may be reflected in the functional properties of dental composites. This problem mostly concerns the surface layer (SL), which is most vulnerable. The temperature gradient is created as a result of heating and cooling of the surface element and the surface and core of the material remaining in the force interaction, inhibit the change of surface dimensions [29]. This phenomenon leads to the formation of the so-called thermal stress of the second type. The temperature distribution in the entire sample volume of the polymer–ceramic composite after thermocycling is shown, among others, at work [30]. In the composites concerned, the polymer matrix is reinforced with a powder filler. The thermal expansion of individual filler particles and polymer matrix is not the same. This results in the formation of thermal stresses of the first type in the PMCCs structure [31], the largest in SL.

Thermodiffusion of water particles to the composite structure may also have influence on SL of dental restorative composites in addition to temperature. The process of diffusion of water particles to multicomponent materials can be described with the help of Fick’s law, which depends on the degree of diffusion from time and temperature. The hydrolytic deterioration of dental composites is believed to intensify in the case of a cyclic load [31]. In addition, the action of stretching thermal stresses causes the opening of microdamages and facilitates the penetration of water [32]. Engineering definition of fatigue is the time dependent fracture of a material due to repetitive cyclic loading [33]. Importance of fatigue for dental restorative composites is clear [15].

Open data on the mechanical and tribological properties and durability of dental materials are limited [34]. These functional properties are important for clinical success [35]. Therefore, the aim of the research is to acquire knowledge useful in the manufacture and operation of medical substitute products for natural-dental composites and comparative study of hybrid and flow types of resin composite materials.

## 2. Experimental Design, Materials, and Methods

The research used light-cured polymer matrix composites (LC PMCCs). Filtek Z550 and Filtek Ultimate Flow composites are commercial materials (3M ESPE, St Paul, USA, MN). Filtek Z550 (Z550, 3M ESPE, St Paul, USA, MN) is a nanohybrid material with a content of 78.5% (by weight) of filler particles. It contains clusters of zirconium and silicon compounds nanoparticles and nanosilica particles. The Z550 matrix is formed by Bis-GMA, UDMA, Bis-EMA, PEGDMA, and TEGDMA resins [36]. Compressive strength Z550 is ≈385 MPa, bending strength ≈150 MPa and tensile strength ≈85 MPa (strength determined by the "Brazilian" method). Z550 is characterized by good abrasion resistance (linear wear of 5 μm/2 × 10^5^ cycles, test in combination of three bodies) [36]. Filtek Ultimate Flow (FFlow, 3M ESPE, St Paul, USA, MN) is a flow composite material, by the manufacturer, 3M ESPE, known as a low viscosity nanocomposite. The FFlow matrix are Bis-EMA, TEGDMA, and Bis-GMA [37] resins. The technical data sheet (TDS) states that the filler is a combination of 0.1 to 5 μm particles and surface-modified silica nanoparticles with dimensions of 20 nm and 75 nm and clusters containing zirconium nanoparticles with main dimensions from 4 to 11 nm and 20 nm silica particles. The dimensions of clusters range from 0.6 to 10 μm [37]. The content of filler particles in the composite is 68% (by weight).

Experimental materials were also used in the research. Experimental material Ex-mhyb(P) is a micro-hybrid composite, the filler of which consists of particles of fluorochar–aluminum–silicon glass, silicon glass, titanium oxide, average particle size: 0.90 μm, filler content 79% (by weight). The Ex-flow(P) material is a flow composite in which the powder filler is made of fluorochar–aluminum–silicon glass, silicon glass, titanium oxide, average particle size: 0.76 μm, the content of filler particles is 64% (by weight). 

Specimens were shaped by a single operator in metal split-mould and then light-cured using light emitting diode (LED) photopolymerization lamp L.E. Demetron (Kerr, Brea, USA, CA, USA) for 40 s impulse.

**Figure 1:**
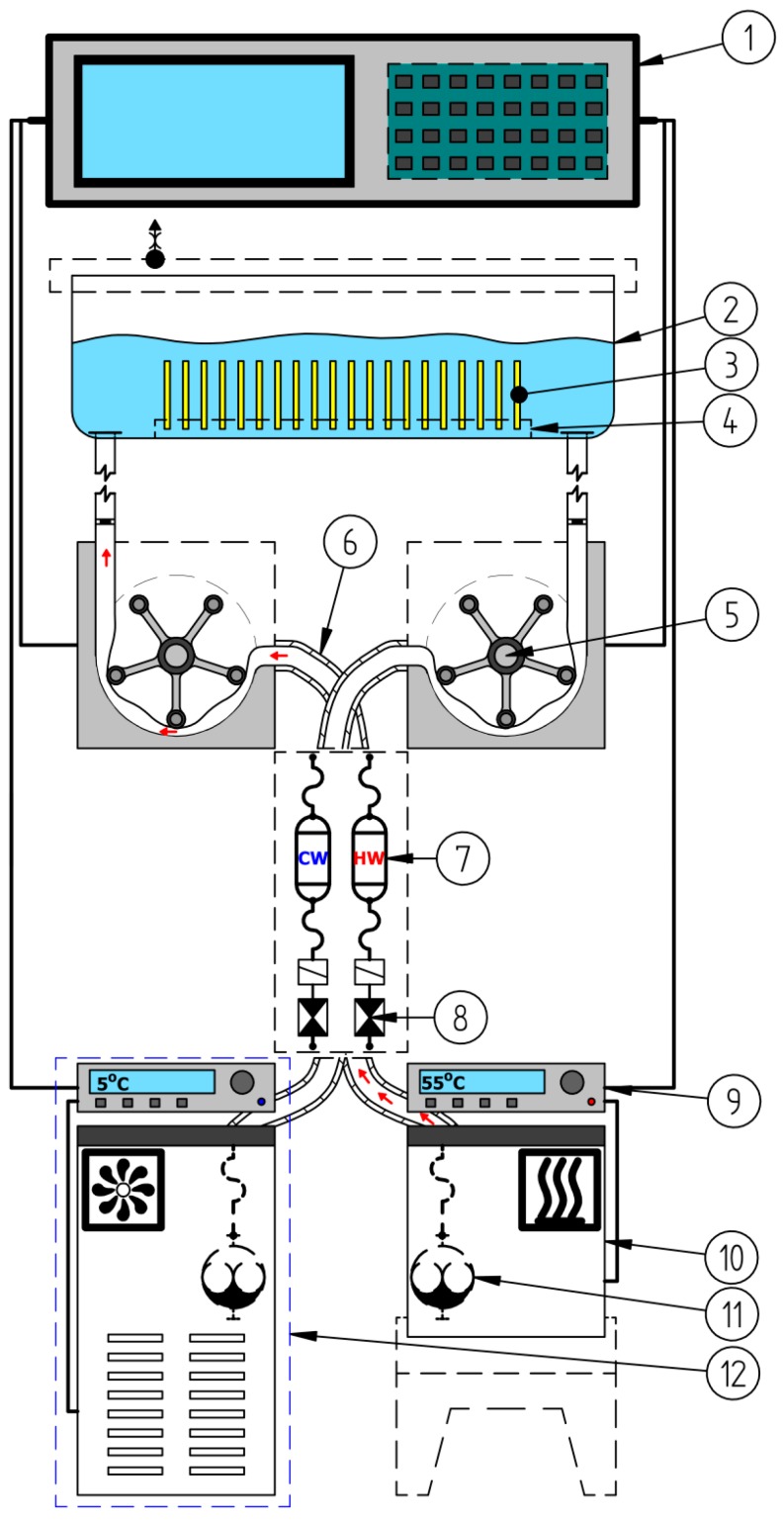
Operation diagram of the device in the pumping phase of heated liquid: 1—digital control system; 2—working vessel; 3—samples on fatigue process; 4—perforated support plate; 5—peristaltic pump; 6—flexible hose insulation; 7—hot water reservoir; 8—electrovalve; 9—thermostat; 10—container with heated liquid; 11—rotary pump; 12—liquid cooling system.

Thermal shock simulations were performed using a thermal shocks simulator designed by the present authors [18]. The simulator (Figure 1) comprised a hydraulic unit and a microprocessor control system. The device produced stepped changes in the temperature of the liquid (water in present study) in the measuring vessel, in which the specimens were immersed. The measuring vessel was alternately filled with heated (55 °C) or cooled (5 °C) working liquid from two separate pumping systems. One cycle lasted 201 s and included: pumping the liquid cooled to the sample vessel (35 s, holding the liquid cooled in the vessel—t_min_ (30 s), pumping out the cooled liquid (35 s), pause (0.5 s), pumping the heated liquid to the sample vessel (35 s), keeping the liquid heated in the vessel—t_max_ (30 s), pumping out the heated liquid (35 s), pause (0.5 s).

Microhardness was measured by the Vickers method using Futertech FM 700 (Future-tech Corp., Kawasaki, Japan) under a load of 0.025 kg. Indenter penetration time was set to 25 s. The tests were carried out at 10 points on the exposed (LC) and non-exposed (NLC) specimen surfaces (LC); the number of specimens in each group was 10 (N = 10). 

The indentation hardness (H) and the elastic modulus of the surface (E) were determined according to the Oliver–Pharr method [38]. The test was performed on the Micro Combi Tester (MCT, Anton Paar GmbH, Ostfildern, Germany). The method is described in more detail in [16,38,39,40].

Wear studies were conducted using a universal microtribometer (Pin-on-Disk Tribometer 0–60 N, CSM Instruments SA, Peseux, Switzerland). The ball-on-disc method was applied. A spherical counterspecimen with a diameter of 6 mm made of aluminum trioxide (Al_2_O_3_, Anton Paar GmbH, Ostfildern, Germany) was used. A constant load of 5 N was used. The testing speed was 60 rpm. The number of specimens in each group was 10 (N = 10). Wear was measured in non-thermocycled specimens and in specimens which had been subjected to 10 × 10^3^ thermal load cycles. There are various wear measurement methods: some measure weight loss [41] while others determine volumetric wear [42]. In this present study, volumetric wear was measured using the Dektak 150 surface profilometer (Veeco, Plainview, USA, NY, USA). All tested specimens were measured on the transverse plane to the sliding direction circle. Distance between profiles was about 35 degrees, 10 measurements were made around the perimeter of the wear scar. The areas of the 2D profiles were integered according to the circumference of the trace of wear which made the determination of the volume of removed material. 

The volumetric wear was calculated on the basis of measurements of the cross-sectional area of the wear path according to the formula

(1) $$V_{disc}=2\pi R\times S_{AR\left(Mean\right)}$$

where:

*V_disc_*—volume wear of a sample from a dental composite (μm^3^),
*R*—radius of wear trace (3 mm = 3 × 10^3^ μm),
*S_AR(Mean)_*—average value of the cross-sectional area of the wear trace (μm^2^).

The wear coefficient was calculated from the following formula [43]

(2) $$K=\frac{HV\left(0.05\right)\times V_{discs}}{F_{N}\times L}$$

where:

HV(0.05)—mean Vickers hardness of the composite (MPa),
*V_discs_*—mean volume loss of the composite (mm^3^),
*F_N_*—normal force (N),
*L*—friction distance (mm).

In addition, specific wear rate *k* was calculated according to [44] using the equation below

(3) $$k=\frac{V_{discs}}{F_{N}\times L}\;$$

where:

*V_discs_*—mean volume loss of the composite (mm^3^),
*F_N_*—normal force (N),
*L*—friction distance (m).

In the work to identify the mechanisms of tribological wear, microscopic studies of the surface of the wear scar were carried out. Observations were made on a scanning electron microscope (SEM) Phenom FEI G2 pro (FEI G2 pro, Phenom-World, Eindhoven, Netherlands). 

## 3. Experimental Results and Discussion

### 3.1. Microhardness

**Figure 2:**
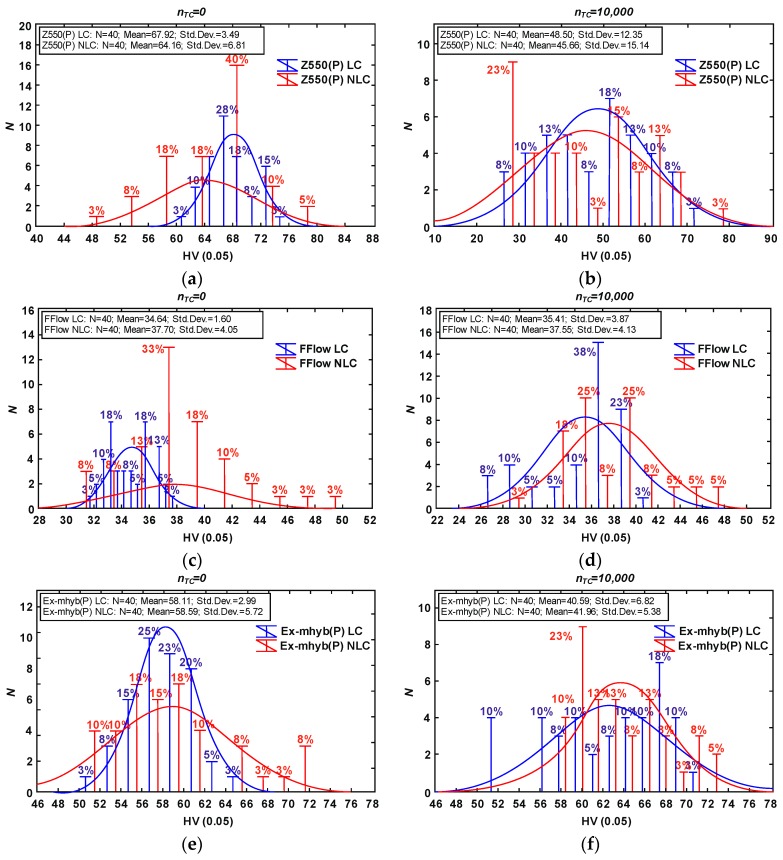

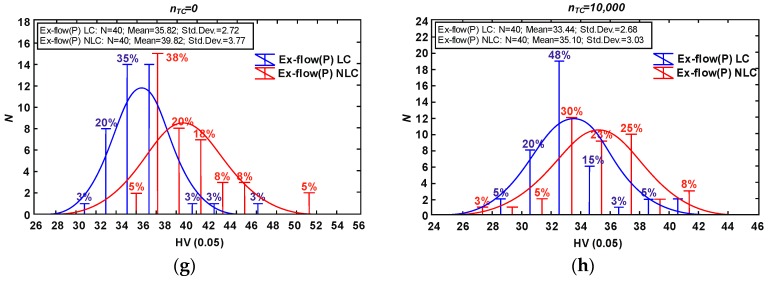
Vickers hardness results due to material type and thermocycling fatigue: Z550 composite (**a**) only aging in artificial saliva (AS), (**b**) after thermocycling (TC); FFlow composite (**c**) only aging in AS, (**d**) after TC; Ex-mhyb (P) composite (**e**) only aging in AS, (**f**) after TC; Ex-flow (P) composite (**g**) only aging in AS, (**h**) after TC. The Vickers hardness diagrams are given in units characteristic for this method (HV).

The results of microhardness tests were compiled on the cardinal histograms (Figure 2). The charts include cardinality, percentage share, and descriptive statistics. The Shapiro–Wilk test of normality (α level of 0.05) was performed (Table 1). The microhardness of the studied composites is varied among materials. The type of composite has an effect on hardness range. Some studies indicate that filler content influences hardness [45,46]. The microhardness of flow composites was lower than for microhybrid materials. Despite the similar content of filler particles in Ex-mhyb(P) and Z550 composites, these materials differed in their hardness. The similar results were obtained by Yeh et al. [47]. In the paper [47], it was found that the proportion of filler particles affects the hardness, however, the effect of the particle size and size of the disperse phase is greater. According to [48], the use of nanoparticles as a part of the filler increases the hardness of the material. According to [49], hardness depends on the size, dispersion, and content of filler particles in the matrix and the adhesion of filler and matrix particles. The degree of cross-linking and conversion can also have a positive effect on microhardness as well as the content of zirconium particles in the Z550 structure. The research presented in [50,51] showed that as the content of zirconium particles in the material increases, the hardness of the composite increases. The hardness of zirconium particles is 17 GPa, while another popular filler, also used in experimental materials, is about 3–4 GPa [45].

The effect of thermocycles on microhardness has been reported in several studies but is not well known. In general, thermocycles cause the degradation and softening of composite resins and consequently decrease mechanical properties [52]. The decrease of microhardness of Z550 i Ex-mhyb(P) composites after thermocycling could be explained by water absorption during cycles. Absorbed water can cause microfracturing of the resin–filler interface and degrade the surface of the filler particles. Similar observations were reported by Tuncer S. et al. [52] who examined resin-based materials after 10 × 10^3^ thermocycles in range temperature from of 5 to 55 °C with a dwell time of 30 s. On the other hand, in the presented study, after thermocycling the mean microhardness volume of FFlow and Ex-flow(P) composites does not change significantly. It might be the result of the heating process occurred during thermocycles. The temperature may have contributed to the post-curing process, further polymerization of the surface, and lead to increased hardness. It is in agreement with the results obtained by [53] in case of Sinfony material but they performed only 5000 cycles in range of temperature from 5 to 55 °C with a dwell time of 30 s. 

### 3.2. Indentation Hardness 

In some works, researchers use the ratio *H*/*E* [54] (indention hardness *H_IT_* to the elastic modulus of the surface *E* based on [38,39,40]) obtained by the Oliver–Pharr method [16]. This coefficient combines the ability to make as much elastic deformation as possible (low modulus) and the ability to minimize permanent deformation (high hardness). For many materials, yield strength *σ_Y_* (yield stress) is related to the hardness *H* (typical *H* *≈ 3σ_Y_*). The *H*/*E* ratio expresses ‘elastic strain to failure’ and elastic resilience of material. The higher the *H*/*E* and *H*^2^/*E* ratio, the greater the wear resistance should be in the material [55,56].

**Figure 3:**
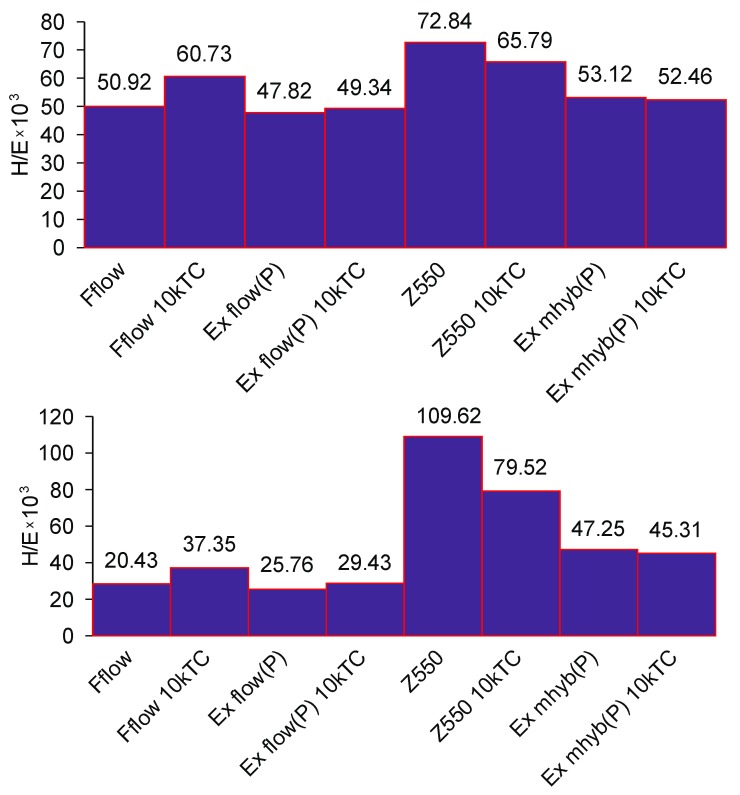
*H*/*E* and *H*^2^/*E* average values for dental composite in relation to thermocycling.

In the tests, the highest value of the *H*/*E* coefficient (Figure 3) is characterized by materials with lower wear resistance (Z550 and Ex-mhyb(P)), the relationships resulting from *H*^2^/*E* comparisons are similar. There is only the dependence of the *H*/*E* and *H*^2^/*E* ratio on the degree of wear inside the material groups determined by the level of the filler content. The direction of variability of *H*/*E* and *H*^2^/*E* coefficients is consistent with the tendency of variability of abrasion resistance due to hydro-thermal fatigue. It seems that the usefulness of the *H*/*E* index in the assessment of the degree of damage may be partial, which is also confirmed by the research presented in [57]. Such dependencies can be explained on the basis of the results of the work [58]. The authors of the study [58] noted that some polymeric materials (e.g., elastomers) show high abrasion resistance, although they are relatively low hard. Similar relations were found for other polymer materials, the paper [59].

### 3.3. Tribological Wear

The wear process, called ‘attrition’, is the most frequent cause of PMCCs wear degradation in the dental biotribological system [60]. Attrition is a variant of abrasion (abrasive) wear occurring as a result of the cooperation of two rigid bodies. Abrasive wear occurs when two rough surfaces cooperate.

#### 3.3.1. Abrasion Testing

**Figure 4:**
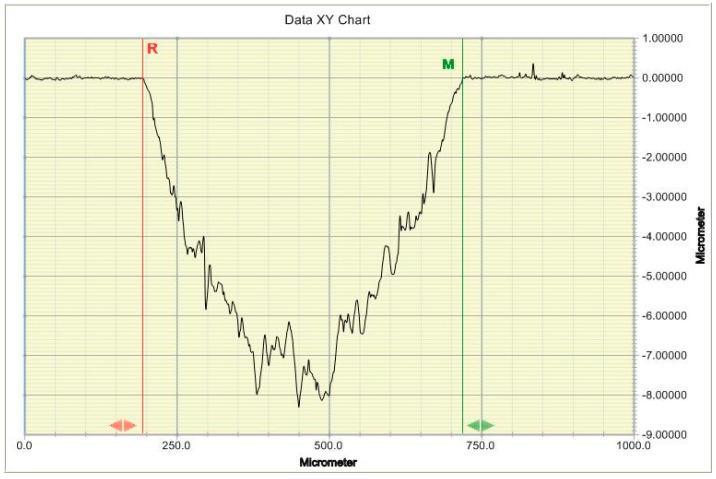
Typical 2D profile of dental material wear mark.

**Figure 5:**
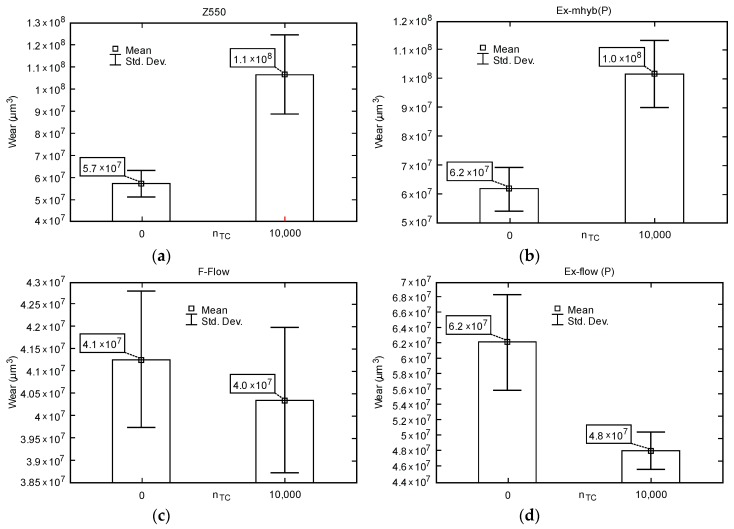
Box-whiskers graphs of wear depending on the number of fatigue cycles TC: (**a**) Z550; (**b**) Ex-mhyb (P); (**c**) FFlow; (**d**) Ex flow(P).

Exemplary traces and wear profiles of the Ex-mhyb composite (P) are shown in Figure 4. Figure 5 presents the results of dental materials wear depending on the thermocycling.

In literature the correlation between the content and the wear resistance of composites was reported. With the increase of filler particles content, wear resistance also increase [61]. However, it was not shown in [62,63,64]. Although FFlow material has lowest grains than Z550 and Ex-mhyb (P), the composite is characterized by the lowest wear among studied materials. The type and size of the filler particles, their dispersion in the material and the adhesion of filler and matrix particles also probably affect the wearing resistance of the composites.

The presented studies show the difference between the results of composites, in case of Z550 and Ex-mhyb (P) materials, after thermocycles. Aging could increase softened surface layer that easily worn out [65]. Moreover, the adhesion of filler and matrix particles may have changed. In the case of ’flow’ type materials, abrasion resistance increased after thermocycling.

Table 2 presents the results of research on the wear of composite surfaces. Wear coefficient (K) and wear rate (k) are included.

**Figure 6:**
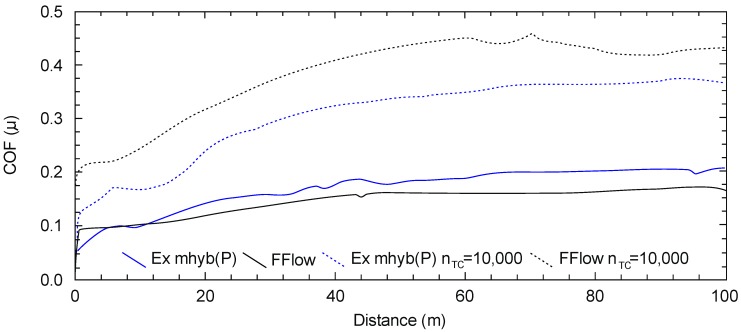
Characteristics of the coefficient of friction (μ) depending on the friction path (dashed line refers to hydro-thermally tired samples).

Table 3 presents the values of friction coefficients. The minimum, maximum, average, and standard deviation of the coefficients of friction are presented. In Figure 6, selected courses of friction coefficients as a function of the friction distance of dental materials are presented. It seems that hydro-thermal non-violent defects overlap with tribological ones, which is manifested by the increased wear of Z550 and Ex-mhyb(P). The phenomenon related to the damage from the hydro-thermal factor is also confirmed by the friction coefficient *μ* as a function of the friction path. The values of the coefficient of friction in the case of Z550, Ex-mhyb(P), FFlow composites increase significantly after thermocycling, which is probably caused by the damage of the surface (Table 3 and Figure 6). However, in the case of a flow-type material, this does not translate into deterioration in wear resistance (Figure 5), which is crucial for durability. In the case of Ex-flow(P) material, thermo-cycling results in a decrease in the coefficient of friction. The phenomenon of increase and decrease in the coefficient of friction of dental composites under the influence of thermocycling was also noted in [30].

#### 3.3.2. Microscopic Images of Traces of Abrasive Wear of Non-Thermocycling Composites

**Figure 7:**
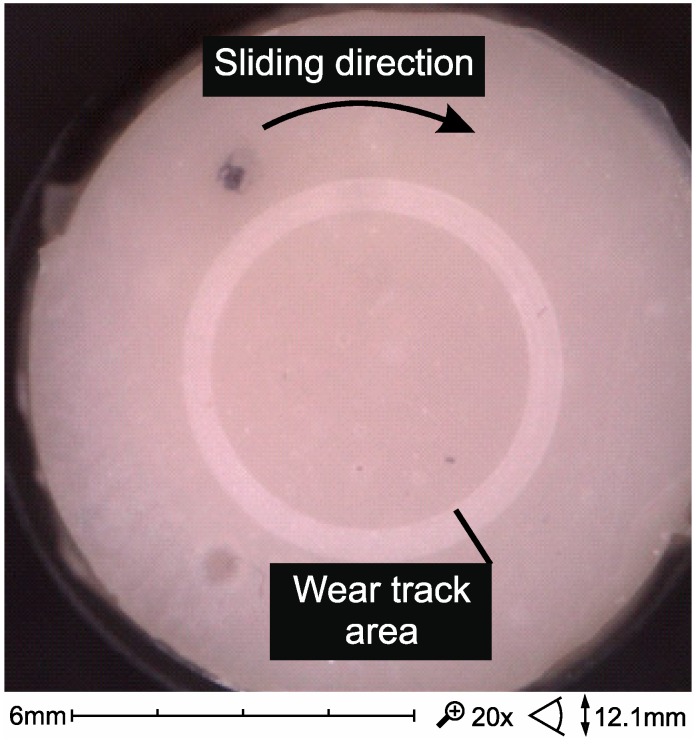
SEM micrographs of wear trace after sliding test against alumina ball in artificial saliva in 37 °C (Ex-flow(P) composite).

The highest wear of dental composites occurs mainly at the point of friction contact and is consistent with the direction of sliding against the sample after the composite sample (Figure 7). In the friction contact zone, there may be solidified or loose abrasive particles [2].

**Figure 8:**
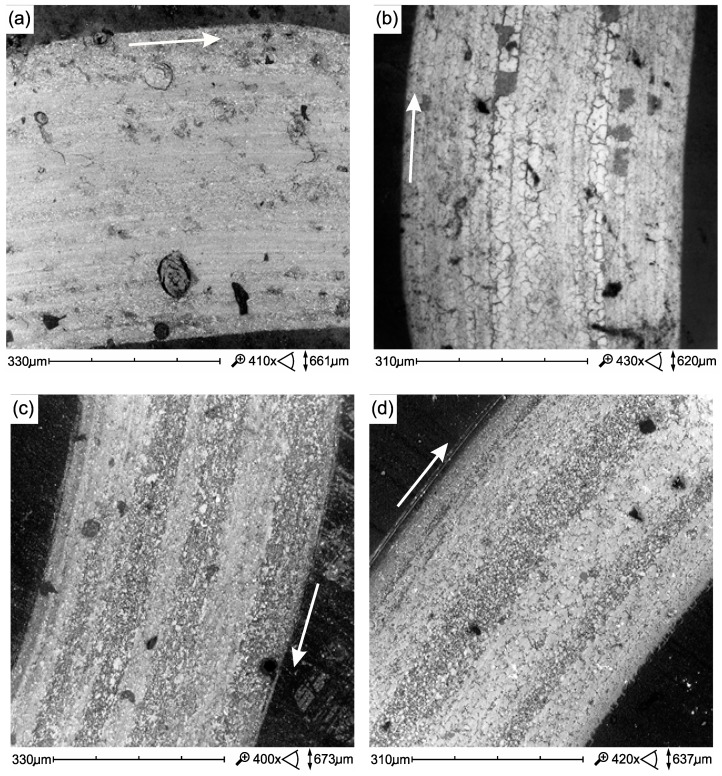
SEM micrographs of wear trace tribological test (samples only aging in AS at 37 °C): (**a**) Ex-mhyb(P), (**b**) Z550, (**c**) Ex-flow(P), (**d**) FFlow. Direction of sliding marked by an arrow.

Figure 8 summarizes the wear trace of the tested composites. Microscars are visible in the images in the trace of wear, consistent with the direction of motion in the kinematic pair, caused by microsurging. Surface defects visible in Figure 8a,b of composites with high filler content are also associated with pitting (a) and spalling (b). In flow-type composites, such damage occurs less frequently (Figure 8c,d).

#### 3.3.3. Microscopic Images of Fatigue Wear 

**Figure 9:**
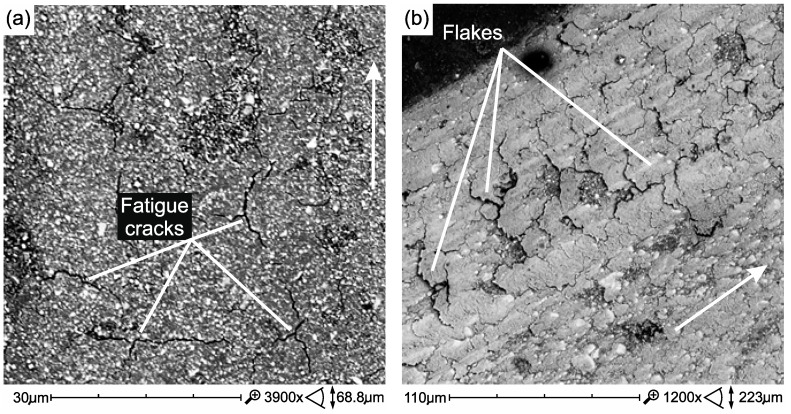
Wear scar of Ex-mhyb(P)—after 10 × 10^3^ TC (**a**), Wear products in the form of flakes, they are symptoms of fatigue wear Z550 after 10 × 10^3^ TC (**b**). Direction of sliding marked by an arrow.

Figure 9a presents fatigue damage of the Ex-mhyb(P) material in the form of microcracks, whose combination leads to the formation of wear debris in the form of so-called material flakes. Our own research confirms that the fatigue wear, often described in the literature, is a typical mechanism of PMCCs surface damage. In [66], it has been shown that as a result of cyclic loads, damage in wear scar subsurface is initiated. Their development towards the surface can cause the removal of large particles-material flakes. To describe this damage mechanism, the model described by Zwierzycki seems appropriate. According to Zwierzycki [67] flake wear, related to the delaminational theory of N.P. Saha, can be described in stages. The flake particle formation stages are as follow: the nucleation of micro-effects, the coalescence of micro-defects into the micro-gap, the propagation of the fracture gap, up to the friction surface, the detachment of wear debris in the form of tiles (flakes) (Figure 9b).

Most of the studies that showed the formation of flaking wear debris were carried out on tribometers in the conditions of sliding friction [66,68,69,70]. Additionally, in studies conducted on chewing simulators, subsurface cracking after mastication cycles was demonstrated [9]. However, other wear mechanisms are also indicated as accompanying the afforementioned issues. Among the mechanisms of wear of PMCCs, abrasion and mechanical fatigue have been indicated [71], mechanical fatigue [72,73], abrasion caused by microcutting and microcracking [74], and delamination [75]. It was found that delamination of composite phases is the predominant mechanism for brittle materials (and these are high-filled PMCCs, K_IC_ stress factor K_IC_ ≈ 1/2 MPa⋅m^0.5^) in the case of relatively large normal loads [76].

**Figure 10:**
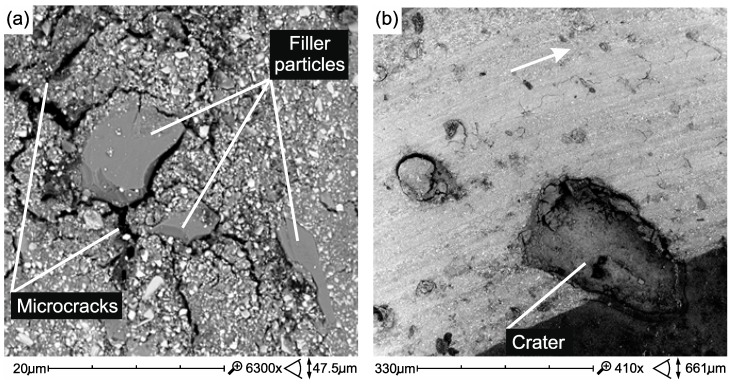
Crushing of large ceramic filler microparticles (**a**) and torn large particles/agglomerates of the filler in wear scar (pitting wear) Ex-mhyb(P) (**b**). Direction of sliding marked by an arrow.

Figure 10 presents damage developing in the environment of inclusions and/or large filler particles. Microcracks initiate in the vicinity of the filler particles (Figure 10a), they develop as a result of cyclic loads, leading to the weakening of the cohesion of the filler particles and lead to chipping (pitting). In Figure 10b, the craters formed after the removal of a large filler particle are visible. Removal of the particle, which was weakly bound due to the development of fatigue cracks, was caused by tacking micro-lines on surfaces cooperating with friction. It is probably an adhesive mechanism of destruction [32].

#### 3.3.4. Microscopic Images of Traces of Abrasive Wear After Thermocycling Composites

**Figure 11:**
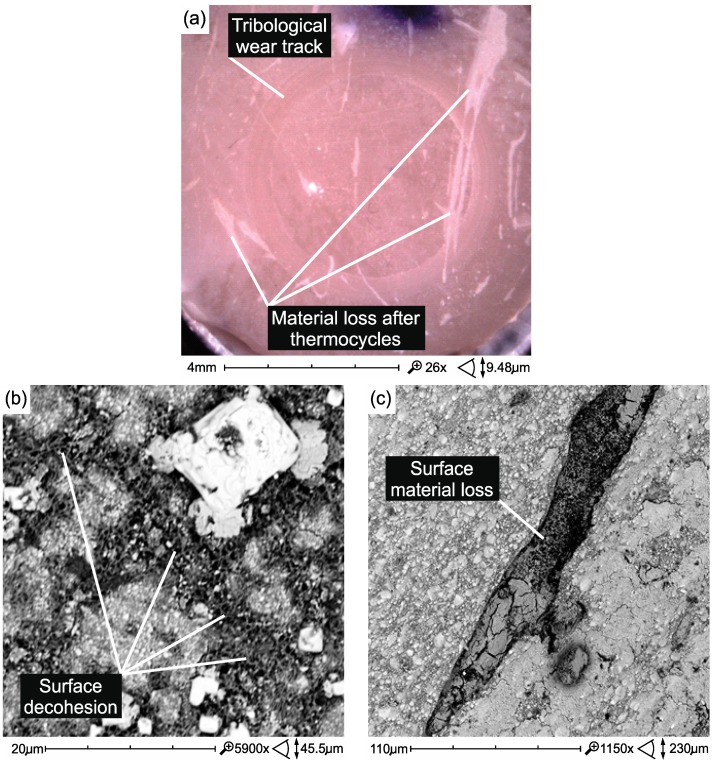
Non-tribological damages accompanying tribological wear caused on the surface of Z550 material samples as a result of thermocycling: (**a**) surface losses of the material and surface roughness increase (Z550); (**b**) surface decohesion (Z550); (**c**) material loss (Z550).

The process of destroying the tribological surface after thermocycling takes place similarly to before thermocycling, but it is more intense. The defects and structure of the structure caused by the process of thermal cycling have a great significance for friction and wear, e.g., areas of surface decohesion and non-abrasive wear (Figure 11a–c)

**Figure 12:**
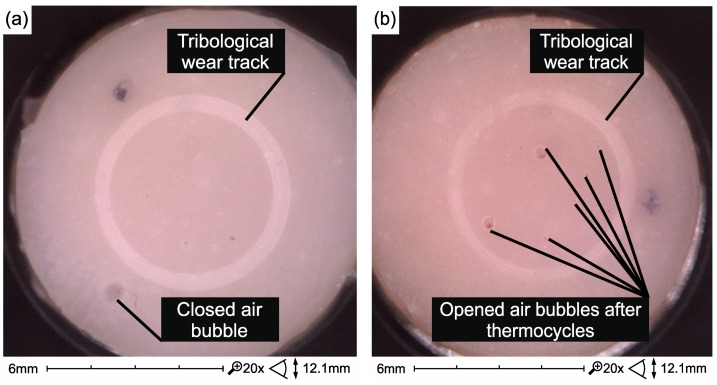
Surface of Ex-flow(P) composite samples without (**a**) and after thermocycling (**b**) and testing on a tribometer.

**Figure 13:**
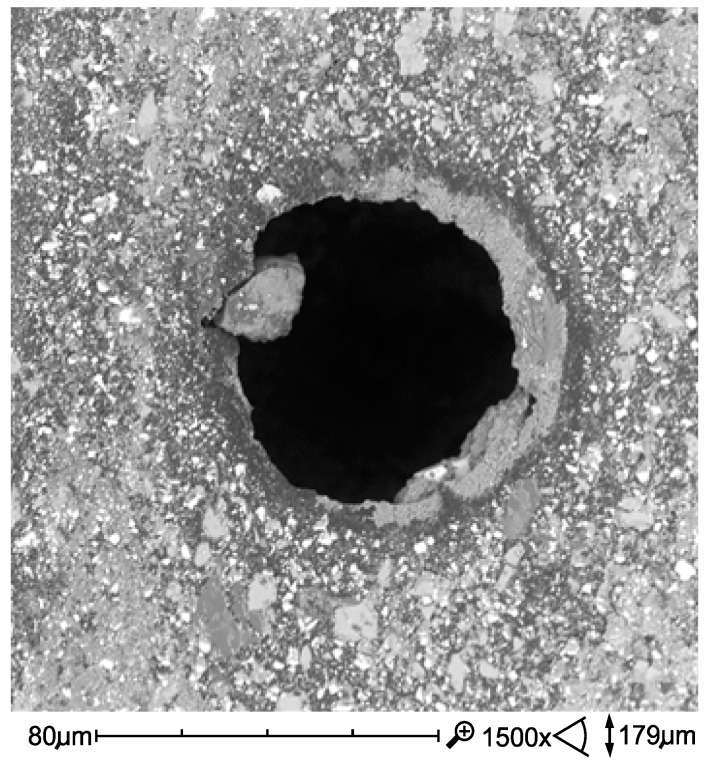
Opened air bubble in Ex-flow(P) composite structure exposed as a result of the combined effect of degradation factors—thermocycling and tribological wear.

In flow composites, surface damage caused by thermal cycling is not so high (Figure 12). However, thermal cycles showed more air bubbles in the structure of the Ex-flow composite (P) (Figure 13).

**Figure 14:**
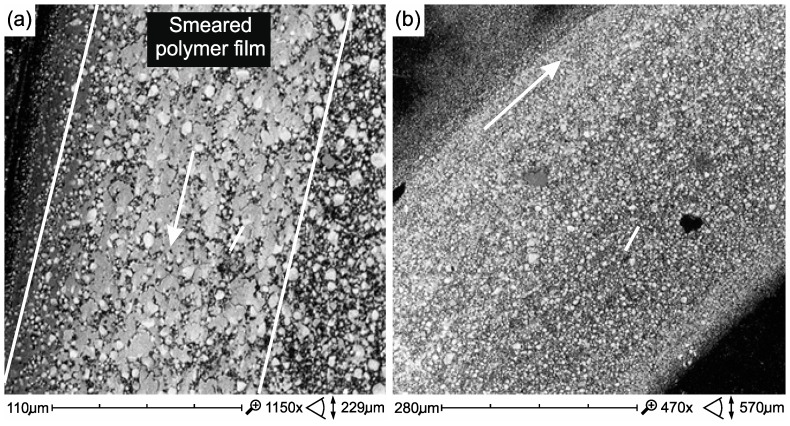
The friction surface of FFlow composite samples without (**a**) and after thermocycling (**b**). Direction of sliding marked by an arrow.

**Figure 15:**
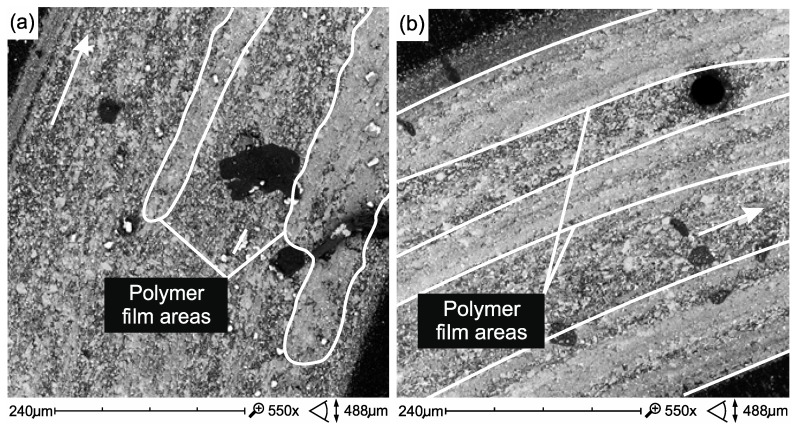
Surface of friction of Ex-flow(P) composite samples without (**a**) and after thermocycling (**b**). Direction of sliding marked by an arrow.

In the case of FFlow before thermocycling, the polymer phase was smeared on the surface of the harder filler particles (Figure 14a). However, after thermal shocks, the formation of a polymer film is less noticeable (Figure 14b), which is most likely reflected in the friction coefficient (Table 3). A reverse relation was observed in the Ex-flow(P) material. The polymer film is more noticeable in the after thermocycling material (Figure 15) which may affect the coefficient of friction (Table 3).

## 4. Conclusions 

The aim of the work was to assess the influence of cyclic hydrothermal interactions on the properties of the surface layer of two micro-hybrid composites and two types of flow composites used as dental restoration materials.

Within the limits of this study, it could be concluded that:

1. The flow composites tested, containing a smaller amount of filler, retain the stability of the mechanical and tribological properties of the surface layer after thermocycling. Materials with a higher filler content (Z550, Ex-mhyb (P)), more strength and hardness, potentially more resistant to wear, proved to be less stable under these conditions.

2. The conducted research allows to better define the mechanisms of tribological and non-tribological mechanical wear.

3. In slip pairs loaded dynamically, under mixed friction conditions, tribological wear of PMCCs, of primary importance for the degree of surface destruction, is manifested by spalling (flaking of the material, in particular polymer phase), pitting (crushing wear caused by wear products), in particular large filler particles or clusters, previously taken apart.

4. On the basis of the obtained results, it should be recognized that in actual kinematic nodes, the exploitative top layer is exposed to significant non-tribological (erosive, thermal, and chemical) damages that are synergistic with the tribological one.

## Figures and Tables

**Table 1:** Shapiro–Wilk test values for normal distribution for Vickers hardness results. Values of p<0.05 are marked in red.

Material	Side	n_TC_	W	p
Z550	LC	0	0.95987	0.16582
Z550	NLC	0	0.97887	0.64768
Z550	LC	10,000	0.96545	0.25589
Z550	NLC	10,000	0.93406	0.02190
FFlow	LC	0	0.96040	0.17285
FFlow	NLC	0	0.96289	0.21006
FFlow	LC	10,000	0.87838	0.00048
FFlow	NLC	10,000	0.95224	0.09058
Ex-mhyb(P)	LC	0	0.98045	0.70620
Ex-mhyb(P)	NLC	0	0.92604	0.01199
Ex-mhyb(P)	LC	10,000	0.93154	0.01808
Ex-mhyb(P)	NLC	10,000	0.94048	0.03590
Ex-flow(P)	LC	0	0.85747	0.00014
Ex-flow(P)	NLC	0	0.85916	0.00015
Ex-flow(P)	LC	10,000	0.90563	0.00280
Ex-flow(P)	NLC	10,000	0.98177	0.75460

**Table 2:** Results of friction wear tests.

Material	n_TC_	LC	K	k (mm^3^/Nm)
Z550	0	40 s LED	2.52 × 10^−2^	1.13 × 10^−4^
Ex-mhyb(P)	0	40 s LED	2.35 × 10^−2^	1.20 × 10^−4^
Fflow	0	40 s LED	1.10 × 10^−2^	8.52 × 10^−5^
Ex-flow(P)	0	40 s LED	1.48 × 10^−2^	1.12 × 10^−4^
Z550	10,000	40 s LED	3.31 × 10^−2^	1.96 × 10^−4^
Ex-mhyb(P)	10,000	40 s LED	2.96 × 10^−2^	2.03 × 10^−2^
Fflow	10,000	40 s LED	1.05 × 10^−2^	8.04 × 10^−5^
Ex-flow(P)	10,000	40 s LED	1.23 × 10^−2^	9.80 × 10^−5^

**Table 3:** Test results of friction coefficient

Material	N	Mean	Min	Max	Std.Dev.
	n_TC_ = 0				
Ex-mhyb(P)	10	0.1431	0.1162	0.1679	0.0173
Ex-flow(P)	10	0.1152	0.0851	0.1462	0.0159
Z550	10	0.2199	0.1947	0.2800	0.0305
FFlow	10	0.1524	0.1325	0.1729	0.0130
	n_TC_ = 10,000				
Ex-mhyb(P)	10	0.3087	0.2578	0.3598	0.0311
Ex-flow(P)	10	0.0614	0.0460	0.1008	0.0186
Z550	10	0.3832	0.2444	0.5132	0.0935
FFlow	10	0.3471	0.2728	0.4368	0.0646
